# Heart Rate Variability, Risk-Taking Behavior and Resilience in Firefighters During a Simulated Extinguish-Fire Task

**DOI:** 10.3389/fphys.2020.00482

**Published:** 2020-07-10

**Authors:** Rebecca Prell, Oliver Opatz, Giampiero Merati, Björn Gesche, Hanns-Christian Gunga, Martina A. Maggioni

**Affiliations:** ^1^Charité – Universitätsmedizin Berlin, Corporate Member of Freie Universität Berlin, Humboldt-Universität zu Berlin, and Berlin Institute of Health, Institute of Physiology Center for Space Medicine and Extreme Environments Berlin, Berlin, Germany; ^2^Berlin Fire Department, Berlin, Germany; ^3^Department of Biomedical Sciences for Health, Università degli Studi di Milano, Milan, Italy; ^4^IRCCS Fondazione Don Carlo Gnocchi, Milan, Italy

**Keywords:** autonomic nervous system, autonomic modulation, heart rate variability, risk-taking behavior, resilience, cardiac stress, workload, firefighters

## Abstract

Firefighters face a high-risk potential, thus their psychological ability to cope with critical or traumatic events is a crucial characteristic. This study examines correlations between cardiac autonomic modulation, risk-taking behavior, and resilience in professional firefighters. Twenty male professional firefighters underwent a 20 min beat-to-beat heart rate (HR) monitoring at baseline in the morning upon awakening, then before, during and after a realistic deployment in a container, systematically set on fire. Risk-taking behavior, resilience, and subjective stress were assessed by specific validated tools after deployment: the *Risk-taking Scale* (R-1), the *Resilience Scale* (RS-13), and the multi-dimensional *NASA-Task Load Index*. The cardiac autonomic modulation at rest and in response to stress was assessed by classic indexes of heart rate variability (HRV) as RMSSD and LF/HF ratio. Results showed that: (i) risk-taking behavior correlated with a withdrawal in vagal indices, shifted the baseline sympathovagal balance toward sympathetic predominance (LF/HF ratio *r*(8) = 0.522, *p* = 0.01), and increased mean HR both in baseline and during physical exercise (*r*(8) = 0.526, *p* = 0.01 and *r*(8) = 0.445, *p* = 0.05, respectively); (ii) resilience was associated with higher vagal indices (RMSSD *r*(18) = 0.288, *p* = 0.04), and with a baseline sympathovagal balance shifted toward parasympathetic predominance (LF/HF ratio *r*(18) = −0.289, *p* = 0.04). Associations of risk-taking behavior and resilience with cardiac autonomic modulation could be demonstrated, showing that HRV may be a valuable monitoring tool in this specific population; however further studies are warranted for validation.

## Introduction

Saving lives under adverse environmental conditions with consciously endangering one’s own well-being, is a constant challenge for the fire service. The working environments of firefighters are characterized by extremely demanding conditions, such as disaster missions, rescue services with an uncertain outcome, as well as firefighting in extremely hot and hostile environments. The working hours are irregular and obligations, such as the recovery of injured or dead people, encumber the mental and physical constitution. Hardly any other job requires a corresponding physical condition and a stable mental state.

Studies mentioned that the typical workload of the task forces correlates with an increased risk of chronic lung diseases (Guidotti, 1995; Weiden et al., 2010; Weakley et al., 2016), cardiovascular diseases (Vrijkotte et al., 2000; Koh et al., 2005; Al-Zaiti and Carey, 2015; Hunter and Mills, 2017), and cancer (LeMasters et al., 2006; Burgess et al., 2018), but also results in mental diseases, such as Post-Traumatic Stress Disorder (Haslam and Mallon, 2003; Blackburn and Owens, 2016; Flatten et al., 2016; Quan et al., 2017; Lee, 2019).

The willingness of firefighters to expose themselves to potentially deadly situations cannot be viewed separated from its psychological dimension. In the past, bravado behavior was postulated, which led to the public perception of a masculine and heroic image of the firefighters (Ericson, 2014). Risk-taking behavior determines the situational awareness, decision-making and the selection of actions—patterns of behavior that affect the interaction and team performance (O’Neill and Rothbard, 2017). Therefore, and because risk-taking behavior also leads to an increased sense of invulnerability (Greene et al., 2000), critical situations can arise. Nevertheless, this circumstance has received relatively little attention so far.

It becomes clear that firefighters face a high-risk potential, which has to be minimized by appropriate training programs. These programs often involve a realistic deployment simulation in a container which is systematically set on fire and has to be deleted from the inside out. Such exercises are tactically adapted to real deployments and provide the unique possibility to observe human behavior and physical adaptations in controlled surroundings.

A further feature to be considered is resilience, defined as the ability to cope with crises in the life cycle by using personal and socially mediated resources and to use them as an opportunity for development (Welter-Enderlin, 2006; Southwick and Charney, 2018). In this sense, resilience has to be understood as a multidimensional, dynamic, and compensatory process that varies over the life span and is strongly depending on the situational and individual state. For employees of fire departments, who face potentially traumatic situations every day, resilience is a profound mental advantage for work ability and quality of life.

Unfortunately, personality traits such as risk-taking behavior and personality states such as resilience are traditionally assessed by self-reports and questionnaires, methods that are prone to reporting biases. A solution to this problem is the introduction of standardized and validated physiological and biological predictors (biomarkers) that allow an objective assessment of both physical and mental processes. The heart rate variability (HRV) is considered a reliable instrument for detecting modulation of heart rate (HR) by the autonomic nervous system (see Task Force of the European Society of Cardiology, 1996; Salters-Pedneault et al., 2010; Minassian et al., 2014), and in the broadest sense a tool to measure stress and stress responses to different stimuli. This tool has successfully been used in to evaluate the human social behavior (Schmidt and Fox, 1994; Tang and Schmidt, 2017). Such measurements are easy to perform and completely non-invasive, and therefore find application in many fields.

The primary aim of this work was to assess whether HRV can be used as a reliable marker for risk-taking behavior and resilience in a group of professional firefighters. The secondary aim was to evaluate the conditions of measurement of this parameter that best predict the psychological attitude to face dangerous situations for these workers (at rest, pre-, during, and post-exposure to simulated stressful conditions of typical working risk and under varying physical workload). Finally, we aimed at observing whether the (possible) additional effect of physical workload during psychological stress might influence HRV.

## Materials and Methods

### Subjects

The sample included *n* = 20 male firefighters in active fire service. All participants were members of the professional fire brigade of Berlin. The average age of the sampled firefighters was 37.8 ± 10.9 (*m* ± SD) years, ranging from 20 to 59 years. Socio-demographic and work-related data were collected for all participants (see Table 1).

**TABLE 1:** Socio-demographic characteristics of the sample.

		*N* = 20
		*n*
Service experience in years	5	4
	6–10	11
	11–20	0
	>20	5
Weekly sports in hours	None	1
	1 h	5
	<1–3 h	6
	More than 3 h	8
Smoking	Never	5
	Quit smoking more than a year ago	7
	Quit smoking less than a year ago	2
	Yes	6
Alcohol consumption per month	Never	1
	Rare (1–4x/month)	8
	Sometimes (5–10x/month)	6
	Frequently (every third day)	5
Drug therapy	None	20

### Psychometric Questionnaires Administration

To collect the psychometric data, three questionnaires were used. The risk-taking scale (R-1) by Beierlein et al. (2014) measures the general willingness to take risks with only one item, in the format of a seven-level rating scale with the poles (1) “not at risk” to (7) “very willing to take risks.” The test results are evaluated according to age, since risk-taking behavior is subject to minor changes during lifetime (Josef et al., 2016). Average values of 4.62 ± 1.39 on the 7-points scale (where the maximum is 7) were found in the standardization for male participants aged 18-35 years reported by Beierlein et al. (2014). Participants aged 36–65 years reached values of 3.76 ± 1.56 and the group of 65 years and above values of 3.51 ± 1.63 (Beierlein et al., 2014). If a person reaches higher values than the age-specific reference, that person can be expected to be more willing to take risks.

For the assessment of resilience, the Resilience Scale (RS) of Wagnild and Young (1993) was selected. The objective of the RS is to capture the internal personal resources and their contribution to the positive resolution of critical life events. The short version with 13 items (RS-13) by Schumacher et al. (2005) allows to recognize persons who are more heavily burdened than other. Similar to the R-1, the answer format is presented on a seven-level rating scale, comprising statements ranging from (1) “completely agree” to (7) “do not agree,” so the highest possible score is 91. According to information of standard values by Schumacher et al. (2005) ranks are determined. A score between 13 and 66 can be considered as a low expression of resilience. A score between 67 and 72 can be considered as moderate expression and a score between 73 and 91 as a high expression of resilience. According to Schumacher et al. (2005) the average for resilience is around 70 ± 12 points.

In order to assess the subjective stress during fire contact the multi-dimensional NASA-Task Load Index (NASA-TLX) by Hart and Staveland (1988) was selected, since it has proven to be sensitive for the measurement of different stress levels of a specific task (Nygren, 1991). The NASA-TLX measures subjective stress during processing a working task on six scales, each with a different dimension. These dimensions relate to features of the task (mental, physical, and temporal demand), behavioral characteristics (performance and effort) and individual characteristics (level of frustration). The scales are bipolar with the verbal description of the minima (0) and maxima (20) at the beginning and end points on the scale, so the highest possible score is 21. Following Rubio et al. (2004) the values of all six scales were added and subsequently divided by the number of scales to get an unweighted mean across all scales. Furthermore, all six scales can also be considered individually, to achieve statements about domain-specific stress.

### HRV Assessment

As for baseline measurement, subjects were instructed to record their early morning HR immediately after waking up at home. The participants were asked to remain in their bed, after waking up, wear the Polar^®^ belt and record HR for 20 min during rest supine. The supine measurement in the early morning immediately after waking up is considered a valid tool for the detection of the short-term cardiac autonomic control in baseline condition at rest, according to the Task Force of the European Society of Cardiology (1996).

As for the measurements before (pre), during (dur) and after (post) a realistic deployment in a container, systematically set on fire, a sitting posture was maintained for all these three 15 min-long measurements; participants were asked to refrain from any food or liquid immediately before the measurements. Further requirements were the abstaining from alcohol and other drugs at least from the night preceding the experiment, as well as to avoid intensive physical training for at least 24 h before the measurement.

RR-interval series were recorded with a validated cardiac monitor (mod. RS800CX^®^, Polar Electro Oy, Kempele, Finland), a system consisting of a chest strap and a watch/training computer as data receiver. The analysis of the HRV indices was performed with the software Kubios HRV ver. 2.1 (Tarvainen et al., 2014). The threshold was set to 0.45 s with the “very low” artefact correction algorithm from Kubios software. Records with more than 3% of beats recognized as artefacts were discarded from the subsequent anaysis. To ensure a better quality of the analysis, only the last 10-min segments at the end of the respective recording period were used. This was done to standardize the measurement, after having analyzed some preliminary data, in which a first transient phase of adaptation to the posture and of relaxation was observed. After this phase, the cardiovascular parameters reached a steady state. The last 10 min were therefore selected manually for each recording file in all measurement points (baseline, before, during, and after deployment in the container). During each bout of exercise, HR quickly reached a plateau value: in those few cases in which HR continued to increase monotonously during exercise, we applied a detrending procedures by subtracting the linear trend of HR increase. HRV indices in time domain (RMSSD and pNN50, both indices of parasympathetic activity), frequency domain (High Frequency—HF—power, index of parasympathetic outflow; low frequency—LF—power, which reflects both parasympathetic and sympathetic activity; LF/HF ratio) and in non-linear domain (detrended fluctuation alpha 1—DFA1—the short-term fractal index, which increases when the vagal outflow decreases or the sympathovagal balance is shifted toward sympathetic predominance) were calculated; see Table 2 for a description of the selected HRV indices.

**TABLE 2:** Selected heart rate variability indices.

Variable	Unit	Description
RMSSD	ms	Root mean square of differences between successive NN intervals. Index of parasympathetic outflow.
pNN50	%	The percentage of successive NN intervals that differ more than 50 ms. Index of parasympathetic outflow.
LF	ms^2^	Low frequency power. Index of sympathetic and parasympathetic activity. Ranges from 0.04–0.15 Hz.
LFnu	nu	LF power in normalized units. LF/(Total Power − VLF) × 100.
HF	ms^2^	High frequency power. Index of parasympathetic activity. Represents the respiratory sinus arrhythmia. Ranges from 0.15–0.40 Hz.
HFnu	nu	HF power in normalized units. HF/(Total Power − VLF) × 100.
LF/HF ratio		Ratio between LF and HF band powers, which represents sympathovagal balance.
DFA1		In detrended fluctuation analysis, short-term fractal index. DFA1 reflects the sympathovagal balance: it increases as the vagal tone decreases.

*NN, normal-to-normal beats, after removing ectopic beats; nu, normalized units.*

### Experimental Procedure

Twenty male professional firefighters underwent a beat-to-beat HR monitoring at baseline (upon awakening a few days before the experiment, 20 min supine), before, during and after a realistic deployment exercise in a container (15 min sitting), systematically set on fire. In order to compare the workload in the container, participants were randomized into two groups. One group (Ex, *n* = 10) performed a physical exercise in the burning container, whereas the other one remained sitting, without exercising (NoEx, *n* = 10).

**FIGURE 1:**
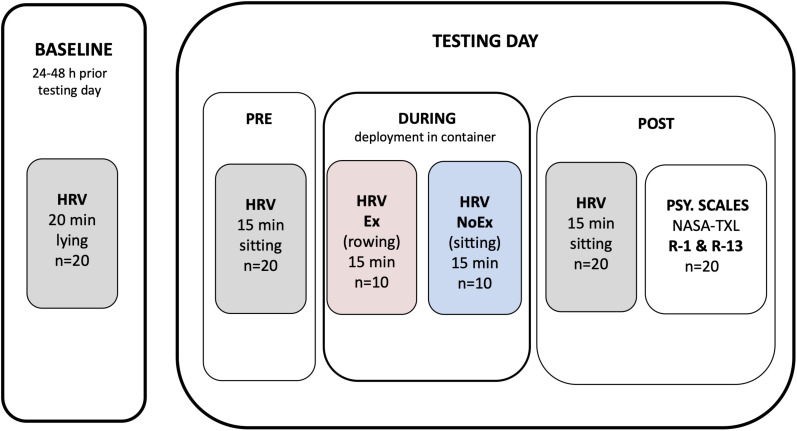
Experimental protocol overview. HRV, heart rate recoding for heart rate variability assessment; PSY. SCALES, psychometric scales conduction.

On the day of the survey, three measure time points were considered. A sitting posture was maintained for all measurements (Figure 1). The measurements without fire contact were carried out in a relaxing area typically used for preparation and instructions. The relaxing area was a covered terrace with benches (ca.15 m^2^), 10 m apart of the container.

Before dressing up with the protective gear, participants were equipped with chest strap systems of the cardiac monitor. The training computer was not worn on the wrist, since it was found, that the straps of the gloves over the watch band were uncomfortably tight. Therefore, the watch was placed around the suspenders. Afterward the participants prepared for entering the burning container in usual routine. For safety reasons, the participants were accompanied by an instructor. In order to enable the instructor to monitor the exercises and to keep his workload as low as possible three participants—and four on the last run—completed the exercises in the container at the same time.

15 min preliminary measurement while sitting in the relaxing area was done immediately before entering the container.

**FIGURE 2:**
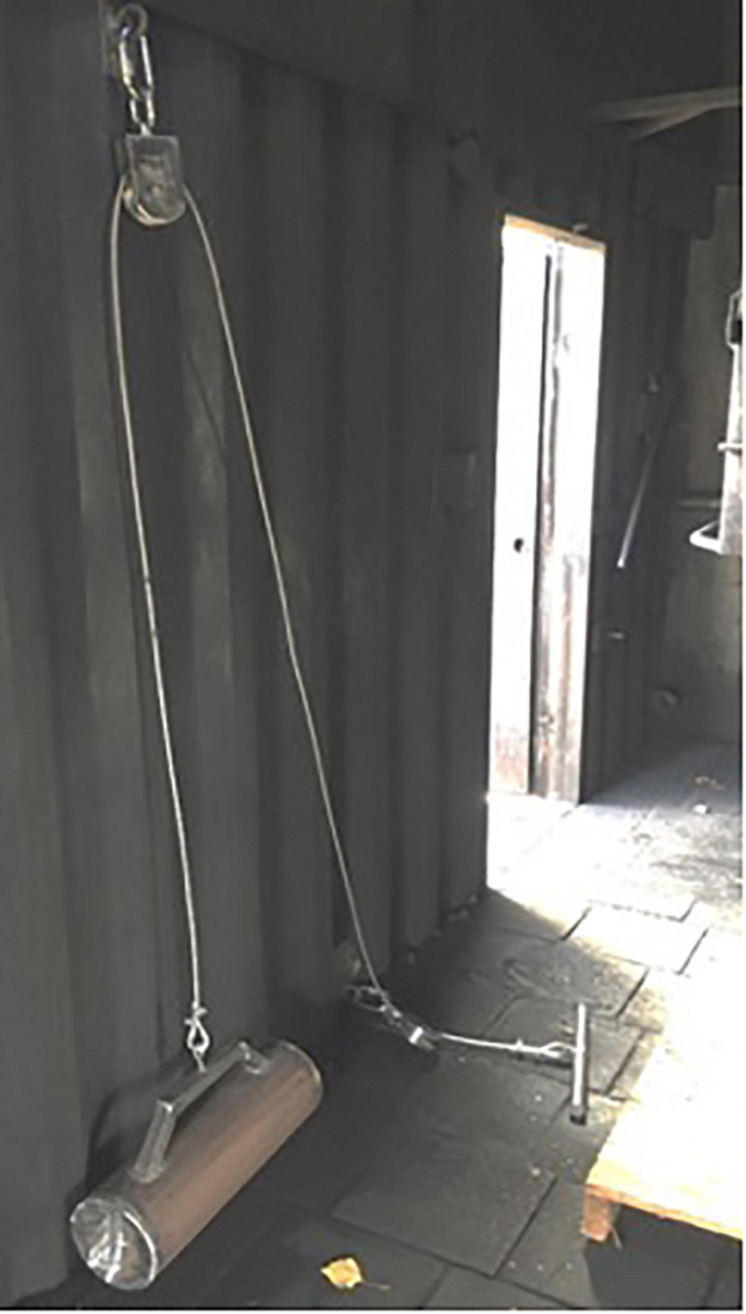
Self-built rower ergometer. A cuff was attached so that the handle could not be pulled further than 60 cm. The wooden pallet identifies the standardized seating position.

The Ex and NoEx groups were given different tasks in the container. The participants of the NoEx group should preferably not move while sitting on wooden pallets and behave calmly inside the container. The Ex group underwent a physical exercise with a home-built rowing ergometer (Figure 2). A 26 kg weight was attached to the cable, to simulate, within the limited space inside the container, the average physical workload of a realistic fire extinguishing task.

**FIGURE 3:**
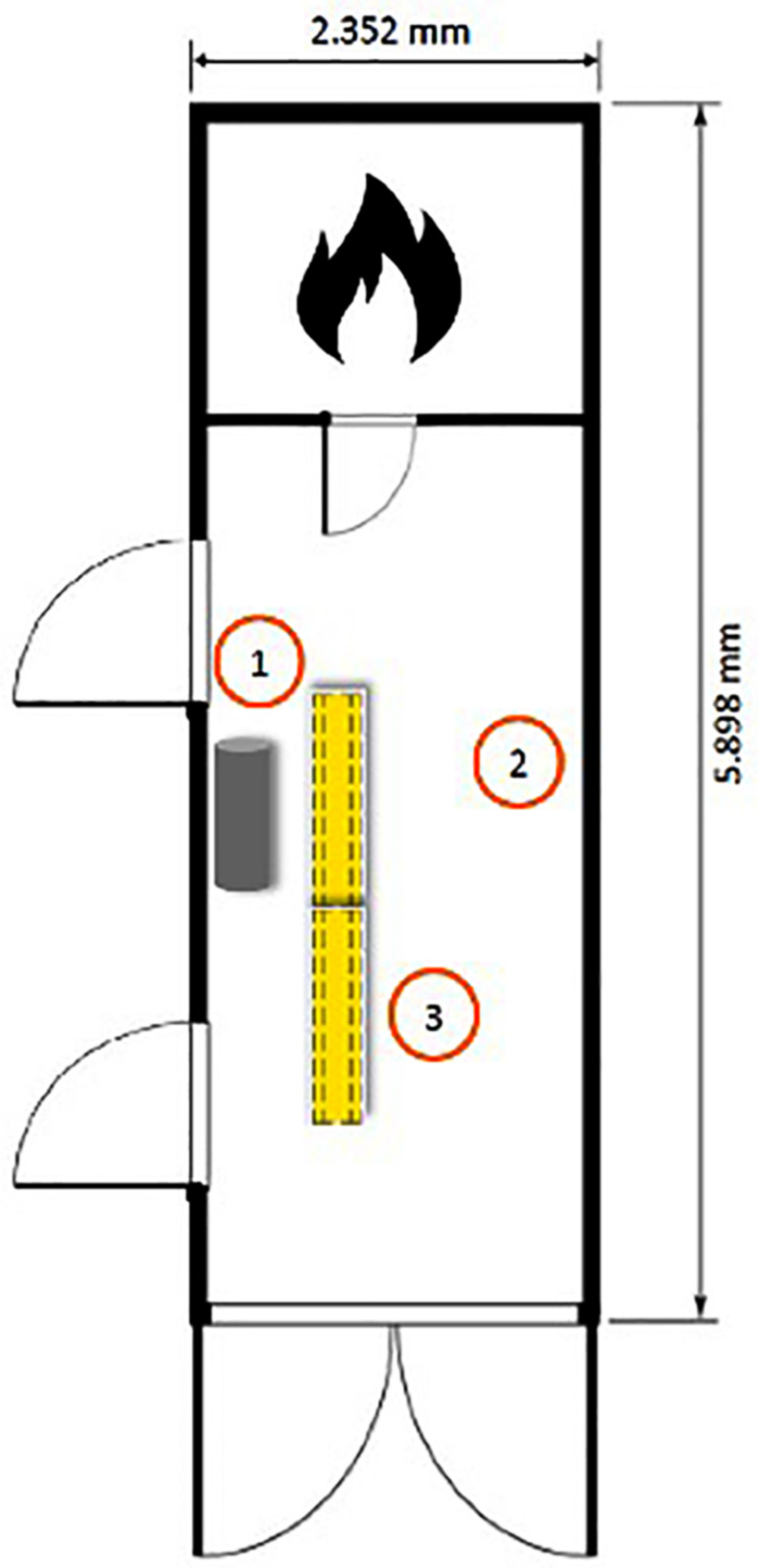
Floor plan of the container. The doors are closed during the exercise. 1. location of the rowing machine, in front of it two stacked seats. 2. location of the experimenter. 3. waiting area for participants.

The general process inside the container was the same for both groups but differed in task definition. The container was set on fire by a study employee in advance. The temperature was achieved by the controlled burning of wood inside a separate booth of the container (Figure 3). The combustion was controlled so that the temperature did not exceed 150°C at 150 cm above the ground (roughly at head level while sitting). By reaching this target temperature the participants were accompanied by the instructor into the burning container. They took their places on the wooden pallets in a sequential order. After all participants had taken seat, a 10 min adaptation phase to the heat in sitting posture was provided. At the end of this phase, a 3 s jet of extinguishing water (8 ± 2 L) was released into the cabin to adjust fire intervention. The relative humidity approached 100%, which is a common phenomenon in realistic firefighting. After this first extinguishing attempt, the actual exercise for the participants of the Ex group began, while the NoEx group remained sitting calm.

The exercise protocol (Ex group) consisted of 10 rowing trains in five sets. When the first participant finished with his first set (10 arm pulls), he slid on the wooden pallets backward and the next participant started to pull his first set. This was iterated five times, so each participant completed a total of 50 arm pulls. Afterward a 3 s jet of extinguishing water was delivered to the container by the instructor and the participants again completed five runs with the same amount of arm pulls as before. Accordingly, the completion of 100 rowing trains was the exercise goal, 50 trains in dry and 50 trains in damp heat. If a competitor did not manage the moves, he was dismissed by the instructor and if necessary, had to leave the container. An average stay of 16.5 ± 0.2 min in the container was recorded with a stopwatch.

After the practice in the container, the procedure of cooling down was prescribed for both groups. The respiratory protection devices were removed and a 15 min recovery phase while sitting in the relaxing area was performed. Subsequently, the participants were asked to complete the NASA TLX, as well as the psychometric questionnaires R-1 and RS-13.

### Statistical Data Analysis

If not otherwise stated, data are reported as mean ± standard deviation (*m* ± SD). The statistical analysis was performed with the statistics program R ver. 3.5.2. All data was tested on homoscedasticity (Levene test), sphericity (Mauchly test) and normal distribution (Anderson’s Darling test). A logarithmic (log) transformation was applied to frequency domain indices to achieve normal distribution (Castiglioni et al., 1999). As the main outcome of this work was the relationship between HRV and resilience parameters, the sample size calculation has been applied *a priori* to the simple linear regression between a representative parameter of resilience (RS-13) and a representative index of parasympathetic tone (RMSSD) in a small preliminary sample of our subjects. The sample size estimate (power of 80%, two tails *t* test on linear bivariate regression, a error 0.05) was of 21 subjects (calculated actual power = 0.81).

A repeated multifactorial ANOVA was used to assess the time course of the cardiovascular changes within and between the subjects. *Post hoc* tests according to Tukey HSD were calculated to discriminate between time points and groups. Linear regression analysis was performed to calculate the correlation between the HRV and psychological data. The significance level was set at *p* = 0.05 with confidence interval at 95%.

Due to the standardization of the Risk Scale (R-1), the participants were categorized in age groups. In the present sample, the first age group (18–35 years) consisted of *n* = 10 people and the second age group (36–65 years) also consisted of *n* = 10 people.

## Results

### Time Course of HRV Indices

**FIGURE 4:**
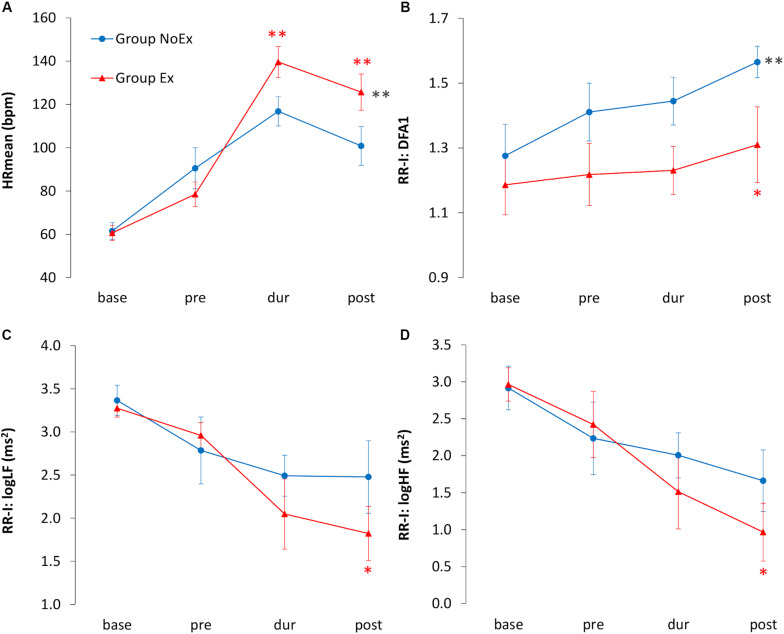
Time course of HRmean and HRV indices. Blue circles indicate the no-exercise group (NoEx) and red triangles indicates the exercise group (Ex). Time of measurements: base, 2 ± 1 days before deployment exercise (baseline); pre, before entering the container; dur, during deployment in the container; post, immediately after deployment. **(A)** HRmean (bpm); **(B)** DFA1 detrended fluctuation alpha 1, index of sympathovagal balance in non-linear domain; **(C)** log LF (ms^2^) low-frequency power, index of sympathetic and parasympathetic drive; **(D)** log HF (ms^2^) high-frequency power, index of vagal tone. Black asterisks: significance between the groups for each timepoint. Red asterisks: significance between the groups for the respective timepoint. **p < 0.01, *p < 0.05.

Significant differences between the groups in HRmean and in the HRV indices LF, HF and DFA1 could be shown. As expected, the HRmean was significantly higher in the Ex group during (*p* = 0.002; +22 bpm, 95% CI [9.70, 35.82]) and in post—after the deployment (*p* = 0.005; +24 bpm; 95% CI [8.62, 41.16]). Furthermore, the Ex group showed a trend to lower LF (*p* = 0.06; -0.65 ms^2^, 95% CI [-0.04, 1.35]) and HF (*p* = 0.07; -0.69 ms^2^, 95% CI [-0.06, 1.45]) after exercise. DFA1 showed a significant difference overall between the groups (*p* = 0.004) and specifically in post (Figure 4), whereas as for baseline and pre (before entering the container) no significant differences between the groups in all HRV indices were retrieved. Overall, as for RMSSD and pNN50 no significant differences between the groups were found.

### HRV and Workload (NASA-TLX)

As for the features of the task *mental demand* and *temporal demand* of the NASA-TLX, correlations could be found. In the NoEx group pNN50 clarifies a significant proportion of the variance of the model with *mental demand*. Forty percent of the variance of pNN50 (*r*(8) = 0.402; *p* = 0.04) is explained by *mental demand*. Furthermore, the indices RMSSD, pNN50 and DFA1 clarify a significant portion of the variance of the model with *temporal demand*. 51% of the variance of RMSSD (*r*(8) = 0.509; *p* = 0.02), 51% of the variance of pNN50 (*r*(8) = 0.514; *p* = 0.02) and 70% of the variance of DFA1 (*r*(8) = −0.700; *p* = 0.003) are explained by the time demand. In the Ex group the behavioral characteristic *performance* clarifies a significant portion of the variance of the model with the indices HRmean, pNN50 and LF. Fifty-four percent of variance of pNN50 (*r*(8) = 0.536; *p* = 0.01), 65% of variance of LF (*r*(8) = 0.650; *p* = 0.005) are explained by performance. The HRmean, is negatively correlated with performance and accounts for 45% of the variance (*r*(8) = -0.445; *p* = 0.03). For the NoEx group the indices LFnu, HFnu, and LF/HF ratio reveal a significant proportion of the variance of the model. Forty-one percent of the variance of LFnu and HFnu (*r*(8) = 0.410; *p* = 0.04), and 57% of the variance of the LF/HF ratio (*r*(8) = 0.571; *p* = 0.01) are explained by the *performance*. The correlation coefficients of the regression analysis provide no significant variance explanation for the model with the total score of the NASA-TLX for both groups. The same occurs for *effort, physical demand*, and *frustration level*. For the overall score, as well as for *effort*, more variance components were clarified by the NoEx group. For the *physical demand*, as well as for *frustration* significantly more variance was informed by the Ex group.

### HRV and Risk-Taking Behavior (R-1)

In the first age group (18–35 years) the average score for risk-taking behavior was 5.20 ± 0.42 (*m* ± SD), which tends to be higher (*p* = 0.10) than the reference sample. Subjects in the second age group (36–65 years) scored on average 4.90 ± 0.73, which is significantly (*p* = 0.05) above the value of the age-specific reference sample (for absolute values see Table 3). Therefore, the present sample is more prone to risky behavior than references.

**TABLE 3:** Absolute values of the analyzed psychometric indicators for risk-taking behavior and resilience (measured after deployment in the container).

Groups	R-1	RS-13
NoEx	5	82
	4	84
	5	73
	5	86
	5	64
	5	76
	6	80
	4	74
	5	73
	5	78
m ± SD	4.9 ± 0.5	77 ± 6.4
Ex	4	68
	5	84
	5	71
	6	88
	6	73
	6	77
	5	84
	5	69
	5	67
	5	70
m ± SD	5.2 ± 0.6	75.1 ± 7.6
*p*-value	0.2	0.5

*R-1, Risk-taking Scale (Beierlein et al., 2014); RS-13, Resilience Scale, short form 13 items (Schumacher et al., 2005). *p*-value: between groups.*

The analysis showed positive correlations for risk-taking firefighters and an increased HRmean at baseline (*r*(8) = 0.526; *p* = 0.01), during (*r*(8) = 0.445; *p* = 0.05) and tendentially post exercise (*r*(8) = 0.408; *p* = 0.06). Significant positive correlations in baseline were also found for the LFnu (*r*(8) = 0.353; *p* = 0.05), the LF/HF ratio (*r*(8) = 0.522; *p* = 0.01), and DFA1 (*r*(8) = 0.402; *p* = 0.03). The latter are also significantly increased in risk-taking firefighters before entering the container (pre) [LF/HF ratio (*r*(8) = 0.419; *p* = 0.03) and DFA1 (*r*(8) = 0.562; *p* = 0.02)]. Significantly negative correlations for risk-taking firefighters were found for the RMSSD in baseline (*r*(8) = -0.345; *p* = 0.05) and pre (*r*(8) = -0.445; *p* = 0.05) and in baseline for the HFnu (*r*(8) = -0.353; *p* = 0.05).

### HRV and Resilience (RS-13)

**FIGURE 5:**
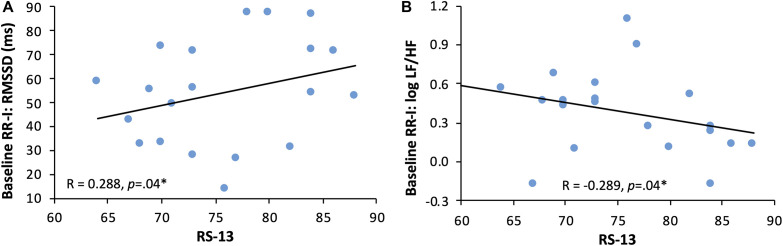
Regression of resilience and HRV indices at baseline. Panel **(A)** RMSSD (ms): root mean square of successive differences between NNs (index of vagal tone) and RS-13 score: short version of Resilience Scale with a score ranging between 13 and 91 (13–66 score: low expression of resilience; 67–72 score: moderate expression of resilience; 73–91 score: high expression of resilience). Panel **(B)** logLF/HF, index of sympathovagal balance in frequency domain and RS-13 score.

**FIGURE 6:**
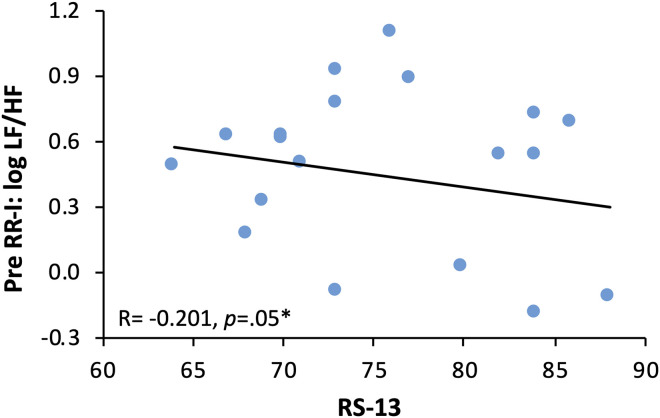
Regression of resilience and logLF/HF at pre. Regression of logLF/HF, index of sympathovagal balance and resilience as RS-13 score (short version of Resilience Scale with a score ranging between 13 and 91 (13–66 score: low expression of resilience; 67–72 score: moderate expression of resilience; 73–91 score: high expression of resilience), before entering the container.

In the present sample, a mean value of 75.76 ± 6.92 of RS-13 was retrieved (for absolute values see Table 3), which corresponds to a feature characteristic in the collective lying above the range reported by Schumacher et al. (2005). Significantly positive correlation could be found for RMSSD (*r*(18) = 0.288; *p* = 0.04) (Figure 5A). Significant negative correlations were retrieved for logLF/HF measured in the baseline and resilience (*r*(18) = -0.289; *p* = 0.04) (Figure 5B) and logLF/HF measured in the pre and resilience (*r*(18) = -0.201; *p* = 0.05) (Figure 6); although not significant, a trend toward a negative relationship between DFA1 and resilience was observed.

## Discussion

In the present study, relationships between mental states and the modulation of the autonomic nervous system in firefighters during a realistic deployment simulation could be shown. The cardiac response to a fire extinguishing task correlated with both physical and subjective stress levels. To the best of our knowledge, the on-the-spot measurement of a real combustion is a hitherto unique setting. The main results of this study can be thus summarized as follows: first, as expected, physical and psychological stress in a high risk environnment typical in firefighting are reflected by cardiac adaptations, secondly, risk-taking behavior is associated with low vagal outflow and thirdly, resilience is associated with high vagal outflow.

Compared to baseline data, a significant shift in sympathovagal balance toward a sympathetic predominance before entering the container could be demonstrated. This means that preparatory actions as well as psychological stress might be measured by the HRV analysis. As expected, in the burning container, the mean HR in both groups is increased, presumably due to a vagal inhibition and/or a sympathetic system stimulation (the neuro-hormonal aspect of catecholamine incretion, although of interest, was beyond the purpose of this study).

After stress, the HRV reflects the dynamic adjustment of the cardiovascular system during the recovery phase and shows a better sympathetic system reactivation in Ex compared to the NoEx group. It can be suggested that physical strain, as expected, is associated with a withdrawal of the vagal indices of HRV, which is also evident in the recovery phase, when the reverse phenomenon can be perceived.

In addition, the exercise appears to affect data collected in the pre with respect to baseline, since preparatory stress displaces the vagal control. Furthermore, as for baseline and pre data, heat stress without physical exercise also results in a reduction of vagal indices, which could be demonstrated in the NoEx group. The assessment of the subjective stress reflects the effect of the exercise on the cardiac response. As expected, more variance proportions are elucidated in task related dimensions, such as *physical effort*. The higher the perceived *physical effort*, the lower the parasympathetic indices, and the higher the *physical demand*, the higher the HRmean. As for the subjective dimension of *performance and frustration*, the exercise’s goal for the Ex group compared to the NoEx group was relatively difficult to achieve. The NoEx group showed significantly positive correlations between parasympathetic drive and *temporal* and *mental demands*. Especially when *temporal demand* was perceived, a sympathovagal balance shifted toward sympathetic predominance could be observed, displaying mental stress processes compared to the Ex group.

In general, the difference between the groups in terms of subjective stress was not as strong as expected. This may have been due to the task sensitivity. The participants of the Ex group were highly motivated to reach the exercise’s goal. If the goal was not achieved, the frustration level was correspondingly high. The participants of the Ex group were only able to attribute the success in the task to their own abilities. On the other hand, the exercise’s goal for the participants of the NoEx group was quite easy to achieve. The only thing they had to do was sitting calm and watching the instructor to extinguish the fire. For this reason, they could attribute elements of the task to the instructor, so an attribution effect could have affected their subjective rating of the NASA-TLX.

It becomes clear that the subjective assessment of workload is reflected in the cardiac stress response and that HRV is a reliable tool to differentiate between stress situations. When considering physiological processes in field investigations, respective environmental factors must be considered. In addition to heat stress, nutrition, caffeine, nicotine and other drugs cause a withdrawal of vagal control (Voss et al., 2015). Beside these environmental and lifestyle-related factors, mental illnesses such as depression or anxiety disorders also result in reduced vagal outflow (Henje-Blom et al., 2010; Liddell et al., 2016). Apart from alcohol, other drugs and drug therapies, these factors were not controlled, even though environmental temperature was standardized during deployment. To our knowledge, there are no data in the literature regarding the effect of such acute and high heat stress on HRV, however, results of the acute effect of sauna on HRV are in line with our findings (Leicht et al., 2018).

Results showed that the personality trait risk-taking behavior is significantly associated with a lower vagal outflow in baseline and pre. In risky firefighters, the HRV indices of parasympathetic activity are significantly reduced. The baseline LF power increased, whereas HF powers and other parasympathetic-related indexes decreased, with the propensity towards risk-taking behavior: this suggest a predominance of sympathetic drive in these individuals. A shift toward sympathetic prevalence is also indicated by the significantly negative correlations between risk-taking scale indexes and logLF/HF and DFA1 at baseline and pre. In addition, for high-risk firefighters, the HRmean is higher in the baseline, during the exercise and in the recovery phase. Thus, there is an association between risk-taking behavior and the positive chronotropic effect of autonomic nervous system on the heart. It can be stated that the personality trait risk-taking behavior in firefighters is associated with the modulation of the autonomic nervous system.

The psychological facets of the sample suggest an above-average level of risk-taking behavior and resilience. Theoretical considerations suggest that risk-taking behavior is particularly pronounced in young adulthood (up to the age of 30 years) (Josef et al., 2016). Although this is also the case in the present sample, older firefighters (35–65 years) are significantly more prone to risk-taking behavior than age-matched reference groups. One reason could be the working environment of firefighters. For example, the subjective risk assessment and the communication of risks between colleagues and within the regulatory structures lead to a reinterpretation of the risk status, which is reflected in the risk attitude and readiness (McLain, 1995; Rötzel, 2015; Buchholz and Knorre, 2019, pp. 177–198). This reinterpretation of the actual risks can lead to an underestimation of the risks and thus lead to a significantly higher willingness to take risks. In addition, the higher level of experience of the older firefighters could lead to increased risk-taking behavior. However, biological, psychological and social changes cannot be ruled out, so that no causality between the willingness to take risks and the fire service profession can be assumed.

Resilience was significantly associated with vagal indices of HRV in the baseline. The hallmark feature resilience is associated with higher vagal indices of HRV. The baseline relationship observed between logLF/HF and RS-13 scores suggests that resilient firefighters have higher parasympathetic control of the heart than less resilient firefighters. However, as expected, this effect is masked during physical activity.

The deployment was simulated, however, there was still a real source of fire, with real fire gases and correspondingly high thematic loads. Underestimating this situation, although widely controlled, leads to fatal consequences. These results support that the HRV may be useful tool for monitoring firefighters’ health and individual fitness not only from physiological point of view, but also for psychological aspects.

### Limitations

The calculated sample size could not be kept due to the utilization of the emergency services, the maintenance and repair of the container and climatic conditions. Women are often under-represented in studies on extreme work environments—especially with the fire departments—which makes the future expansion to female participants more relevant.

Furthermore, we decided to include the LF/HF ratio as a marker of a shift toward sympathetic drive, in spite that the meaning of this index is currently controversial (Heathers, 2012).

The classification of homogeneous fitness, experience and age groups also appears to be useful in the analysis of HRV, as significant differences in linear and non-linear indices have been demonstrated when comparing age and gender groups and the fitness level. Thus, a significant loss of variability and complexity of HRV is associated with sex and age (Voss et al., 2015). Due to the size of the sample, we couldn’t infer any effects of sociodemographic data on the relationship between HRV results and psychometric data, which need to be investigated in further studies.

## Conclusion

On one hand, HRV is a tool to monitor cardiovascular fitness, as health-promoting means, disease prevention and to improve cardiac fitness among firefighters. On the other hand, HRV can be used to assess psychological states as well as personality traits. Knowing how psychological aspects are associated with autonomic nervous system modulation, may allow to integrate new personalised reccomendations, which are of crucial relevance, especially in this specific professional group. Therefore, biomarkers as HRV are key elements of personalized diagnostics, therapy and treatment.

HRV is a particularly beneficial technique because the measurement is relatively inexpensive, comfortable and provides qualitative data, at least intra-individually reproducible.

## Data Availability Statement

The datasets for this article are not publicly available due to privacy protection reasons. Requests to access the datasets should be directed to the corresponding author.

## Ethics Statement

This study was approved by the Ethics Committee of the Charité University of Medicine Berlin (document number EA4/177/17) and by the Head of Berlin Fire Department. All subjects gave written informed consent in accordance with the Declaration of Helsinki (World Medical Association, 2013).

## Author Contributions

MM and RP developed the theory, performed the computations, and verified the analytical methods. RP collected the data. OO conceived the idea and provided the project’s setting. BG organized the work on site and contributed to data collection. GM encouraged to investigate specific HRV indices and supervised the findings of this work. H-CG supervised the project. All authors discussed the results and contributed to the final manuscript.

## Conflict of Interest

The authors declare that the research was conducted in the absence of any commercial or financial relationships that could be construed as a potential conflict of interest.
